# Supplementary material for: Baseline patterns of infection in regions of Benin, Malawi and India seeking to interrupt transmission of soil transmitted helminths (STH) in the DeWorm3 trial
Source: PLoS Negl Trop Dis. 2020 Nov 2;14(11):e0008771. doi: 10.1371/journal.pntd.0008771 (PMC7673551; doi:10.1371/journal.pntd.0008771)
Supplement: S1 Text — (DOCX) [file pntd.0008771.s001.docx]

**Supplementary information**

**Force of Infection**

In the absence of an independent estimator of the egg-to-worm relationship, the force of infection (FOI) defined as the per host rate at which an individual acquires parasites that survive to reproductive maturity in the human host, inferred from the age-egg count data from faecal material assumes that the mean egg count *E* is a direct proxy for the mean worm burden *M* within the human host such that


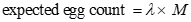


Here, the coefficient λ is the average per worm egg production rate which is assumed to be independent of host age. Such a relationship is justified, especially in the limit of smaller worm burdens; at which point, the small argument expansion of the exponential density-dependent fecundity relationship observed from studies of worm expulsion and egg counts prior to worm expulsion (Ref Anderson and May, 1991) yields in this limit. From this assumption, the procedure to generate the FOI as a function of age is to integrate the equilibrium worm burden transmission equation over age. Therefore, numerical integration approximations provide a method to connect the mean worm burden as a function of age to the FOI. Once a mean worm burden has been inferred for a specific age bin, the expected egg count may be obtained from the first equation and the negative binomial likelihood with this mean and a clumping factor of 1/*k* is used to model the statistical uncertainty surrounding the egg count values.

We further combined the negative binomial likelihood with an ensemble Markov Chain sampler (<http://dfm.io/emcee/current/>) to obtain the inferred parameters for the fits of FOI as a function of age.

**Aggregation from cluster-level prevalence-intensity data**

The degree of aggregation in the distribution of worms among individuals can be approximately inferred from the relationship between the mean egg count and the prevalence in individual clusters. We assume that worm aggregation is uniform across all clusters. As stated in the main text, the relationship between prevalence and egg count intensity is given by


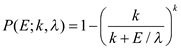


where k is the negative binomial aggregation, λ is the net egg count per fertilized worm and the term
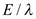
 is based on the same rationale as the first equation of the supplementary information. Using this relationship, a likelihood function can be written for the cluster level baseline data in each country,


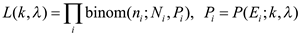


where
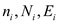
 are the number of positive individuals, the total number of individuals and the mean egg count, respectively, in cluster *i*. The likelihood was used, with flat priors, to estimate the maximum likelihood estimators for the parameters *k* and λ along with their 95% credibility intervals. These are quoted in Table 2 in the main text. The method used here is a simplification and omits two sources of variance which may result in bias. Both arise from the variability in the measured egg count for individuals. These contribute an extra source of uncertainty in both the prevalence and mean egg count in a population. As a result, the method tends to overestimate the aggregation of worms (decrease the value of the aggregation parameter, *k*). Inclusion of these effects is too complex for the current analysis but has been explored elsewhere (DOI: 10.1186/s13071-019-3686-2).

**Total egg count and probability of heavy infection (as defined by WHO) by age**

The estimates of total egg count and probability of heavy infection by age are both based on the same model. A linear model of mean egg count as a function of age was fitted to the full individual-level egg count data from the baseline. The likelihood for the data given the model is


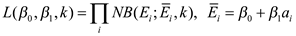


where
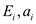
 are the egg count and age of the individual *i* at baseline. Maximum likelihood estimators (mle) for the parameters β_0_, β_1_ and *k* along with their 95% credibility intervals where estimated from the posterior distribution, assuming non-informative priors. The cross-sectional survey was used as representation of the baseline demographic structure for the population and the mle parameter values were used to estimate the mean total egg count in 5-year age groups. Uncertainty in the total egg count estimate, and also the probability of heavy infection, were calculated by resampling parameter values from the posterior distribution of the model fit.
