# Supplementary material for: Baseline patterns of infection in regions of Benin, Malawi and India seeking to interrupt transmission of soil transmitted helminths (STH) in the DeWorm3 trial
Source: PLoS Negl Trop Dis. 2020 Nov 2;14(11):e0008771. doi: 10.1371/journal.pntd.0008771 (PMC7673551; doi:10.1371/journal.pntd.0008771)
Supplement: S2 Text — (DOCX) [file pntd.0008771.s002.docx]

**The DeWorm3 Trials Team Author List**

Abraham Dilip^1^, Ajjampur Sitara SR^1^, Anderson Roy M^2^, Ásbjörnsdóttir Kristjana Hrönn^3^, Avokpaho E^4^, Bailey RL^5^, Cottrell G^6^, Farzana Jasmine^1^, Galagan Sean^7^, Halliday Katherine Elizabeth^8^, Hardwick Robert J^2^, Houngbégnon P^4^, Ibikounlé M^9^, Juziwelo Lazarus^10^, Kaliappan Saravanakumar Puthupalayam^1^, Kalua K^11,12^, Kang Gagandeep^1^, Kennedy David Stephen^8^, Kepha S^8,13,14^, Legge Kenneth Hugo Callum^8^, Littlewood Tim^15^, Luty AJF^6^, Manuel Malathi^1^, Means Arianna Rubin^16^, Oswald William Edward^8^, Palanisamy Gokila^1^, Pullan RL^8^, Simwanza J^11^, Truscott James E^2^, Walson Judd L^3,16,17,18,19^, Werkman Marleen^2^, Witek-McManus S^5^.

^1^The Wellcome Trust Research Laboratory, Division of Gastrointestinal Sciences, Christian Medical College, Vellore, India

^2^London Centre for Neglected Tropical Disease Research, Department of Infectious Disease Epidemiology, School of Public Health, St. Marys Campus, Imperial College London, London, United Kingdom

^3^Department of Epidemiology, University of Washington, Seattle, Washington, United States of America

^4^Institut de Recherche Clinique du Benin, Abomey-Calavi, Benin

^5^Faculty of Infectious and Tropical Diseases, London School of Hygiene & Tropical Medicine, London, United Kingdom

^6^Université de Paris, MERIT, Institut de Recherche pour le Développement, Paris, France

^7^International Clinical Research Center, University of Washington, Seattle, Washington, United States of America

^8^Department of Disease Control, Faculty of Infectious and Tropical Diseases, London School of Hygiene & Tropical Medicine, London, United Kingdom

^9^Département de Zoologie, Faculté des Sciences et Techniques, Université d’Abomey-Calavi ; Institut de Recherche Clinique du Benin, Abomey-Calavi, Benin

^10^National Schistosomiasis and STH Control Programme, Community Health Sciences Unit, Ministry of Health, Lilongwe, Malawi

^11^Blantyre Institute for Community Outreach, Blantyre, Malawi

^12^Department of Ophthalmology, University of Malawi, College of Medicine, Blantyre, Malawi

^13^Pwani University, Kilifi, Kenya

^14^Eastern and Southern Africa Centre of International Parasite Control, Kenya Medical Research Institute, Nairobi, Kenya

^15^Parasites & Vectors, Department of Life Sciences, Natural History Museum, London, United Kingdom

^16^Department of Global Health, University of Washington, Seattle, Washington, United States of America

^17^Department of Medicine, University of Washington, Seattle, Washington, United States of America

^18^Department of Pediatrics, University of Washington, Seattle, Washington, United States of America

^19^Natural History Museum, London, United Kingdom
