# Supplementary material for: Baseline patterns of infection in regions of Benin, Malawi and India seeking to interrupt transmission of soil transmitted helminths (STH) in the DeWorm3 trial
Source: PLoS Negl Trop Dis. 2020 Nov 2;14(11):e0008771. doi: 10.1371/journal.pntd.0008771 (PMC7673551; doi:10.1371/journal.pntd.0008771)
Supplement: Attachment — Submitted filename: PLOS NTD Responses to reviewer comments 2020-07-04_FINAL.pdf [file pntd.0008771.s003.pdf]

deworm3

July 4, 2020

Dear Editors,

We wish to thank the editors for the opportunity to respond to the excellent reviewer comments provided. We feel as though the revised manuscript has been substantially improved. As noted previously, we are confident that these data will be of interest to the readers of PLOS NTDs and we are hopeful that the editors and the reviewers find the revised manuscript acceptable for publication.

On behalf of the DeWorm3 Project Team

Judd Walson, MD, MPH
Principal Investigator, DeWorm3

Response to reviewer comments

This is a baseline-survey report from an important 3-country cluster RCT. As a publication it is sandwiched between the protocol/design article, and the main results article when that article arrives in a couple of years. Such a "middle article" tends to be overshadowed by the other two, but is of course important on its own merit, as well as in "communicating" with them and establishing the overall context of the study, its capabilities and limitations.

- *We thank the reviewer for these comments and we agree that this manuscript is important to establish the context of the study.*

Therefore, it is worth comparing the estimated prevalences with the assumptions of the protocol article. It seems that India where prevalence was presumed highest, is even higher than the assumptions; in Malawi it's right in the 7%-10% range stipulated in power calculations, while in Benin it's somewhat lower.

- *This is an excellent point. We have added a sentence to the Discussion highlighting these factors.*

  "*Baseline prevalence of STH varied substantially across DeWorm3 sites. While prevalence in India was 21.4%, prevalence in Benin and Malawi was <10%, and species-specific prevalence in Benin, the only site with a substantial Ascaris prevalence, was even lower at ~2%. Sensitivity of Kato Katz is low at low prevalence and intensity of infection, meaning that these prevalences are likely to be underestimated[30].*

More broadly, I wonder how well the assumption that <2% means interrupted transmission, holds up vs. the data showing Ascaris and Trichuris are both <=2% everywhere, yet are apparently endemic. Are these two species concentrated among older residents who had not experienced the child MDAs? Regardless, what do you think is happening with these species? Were they more prevalent before and improvements/treatments have driven them to near-elimination, or have they been

endemic this way for a long time - which might throw a wrench into the <2% target underpinning the entire trial? Or is the <2% assumption mostly relevant to hookworms? STH are not my specific field so my questions might have an easy answer. Or could this be related to the reported poor sensitivity of Kato-Katz at low prevalences?

*We thank the reviewer for this comment, as it is an important point. Hookworm is the predominant species at all three DeWorm3 trial sites, while Ascaris is present in some clusters in the Benin site and Trichuris prevalence is minimal. It is important to note that the definition of transmission interruption is dependent not on the prevalence of infection as detected by Kato-Katz, but using molecular methods (qPCR). It is likely that the prevalence of both Ascaris and Trichuris are significantly higher than those reported at baseline using Kato-Katz. When qPCR is complete, we will have a much more accurate sense of the true baseline prevalence at each site and in each cluster. This is especially true in Benin, where in clusters in which Ascaris is present, its prevalence is higher than 2%, although the weighted average prevalence across the whole site is 2.0%. Of note, in areas of Japan, where Ascaris prevalence was exceptionally high in the 1950s and 1960s, reductions in prevalence below 2% as well as substantial reductions in overall intensity of infection by the early 1970's did indeed correspond to the elimination of ascariasis in the population (*Hasegawa et al. Parasites Vectors (2020) 13:6 https://doi.org/10.1186/s13071-019-3875-z*)*

We have added a clarification on this point to the limitations paragraph of the discussion:

> *"First, this paper reports Kato Katz fecal egg counts as measures of the intensity of infection in individuals, rather than by qPCR, the method by which the primary outcome – interruption of transmission – will be assessed at the end of the trial, which has several implications. As mentioned previously, Kato Katz is known to be unreliable at low prevalences of infection, tending to underestimate the true prevalence[16]."*

It is difficult for me to completely evaluate much of the methods and possibly some of the results, because I could not locate the supplementary data file.

- *We apologize for this oversight. The supplementary file is provided along with the revision of the manuscript.*

Major Comments

- Methods first paragraph: There is little sense in naming only the India regions just because they happen to be two, while providing zero details elsewhere. Either provide more complete details (e.g., "In India the site is in a rural part of the southern state of Tamil Nadu, xx km from the nearest major city Y." and likewise for Benin/Malawi), or just refer people to the figure and to the protocol article. I prefer the former: describing the sites a bit more will help understand better the differences in burden patterns you report later, and which are so central to this article. Perhaps a few sentences from the first paragraph in Results are more appropriate here than there.

- *We have updated this paragraph to reflect the contrast between the Malawi and Benin and the India site as follows:*

> *"In both Malawi and Benin, the sites consist of geographically contiguous areas, with the Malawi site located in a rural area of Mangochi district, in the Southern*

region; and the Benin site comprising Comé town and the surrounding rural area in the Commune of Comé. Inrespectively; while in India, the study area consists of two geographically distinct sub-sites within the state of Tamil Nadu - a plains area in, Timiri and a tribal region in Jawadhu Hills."

- In Methods, there are no details about how FOI is estimated, and I could not locate the supplement where details of the aggregation-parameter estimate are found.

- *We apologize that the supplementary materials were not available to the reviewers. We have included these again with the submission and hope that these address the comment.*

- Does the supplement include prevalence-by-age data? I think those data would be useful. Figure 4 shows total eggs per cluster by age, but unless I'm mistaken this is not the same information. Likely even in the main article, at least the prevalence by the 3 age strata for each species and site.

- *We have added panels to Figure 3 to show prevalence by age strata (PSAC, SAC and adults).*

- Also, not sure how the eggs per cluster translate into eggs per person. Or is there a miswording in Figure 4's description?

- *We apologize for this error. "Per cluster" should read "by age", and this has now been corrected. Further details are provided in the supplementary information.*

Minor Comments

- Figure 1: a map of the world is not very helpful here. Makes more sense to have 3 meso-scale maps showing where in each country the study site is (for the smaller countries it might show parts of neighboring countries as well).

- Related, figure 2: I understand that this map is also intended to show all cluster boundaries. But maps are there to provide context, so zooming out slightly (while retaining cluster boundaries) and showing a bit additional information of the surrounding (town/landmark names, nearest town/city outside study district, etc.) should help understand the context, rather than just be a series of black lines and dots against a grey background.

- *We have replaced both Figure 1 and Figure 2 with a single figure showing the location of study sites within the borders of each country. The figures are supplied in both color (in order to provide more detail on the surrounding area) and in grayscale (to facilitate printing).*

- Methods p.11, mention of Kato-Katz. Please provide reference.

- *We have added two references:*

Katz N CA, Pellegrino J. A simple device for quantitative stool thick-smear technique in schistosomiasis mansoni. Rev Inst Med Trop São Paulo. 1972;14:397-400.
Assessing the epidemiology of soil-transmitted helminths during a transmission assessment survey (TAS). Geneva: WHO/Department of control of neglected tropical diseases, September 2015 Contract No.: WHO/HTM/NTD/PCT/2015.2.

- Methods p.11, formulae for prevalence estimates. Not sure the actual formulae are needed since they are pretty straightforward weighted averages, and the text describes what you do well enough.

- *We have made this change.*

- Methods, end of p.11. Please add version of R.

- *We have made this change.*

- Results p.13, assets and living conditions. It is always a challenge what details to place in the body text before it becomes a laundry list. I suggest providing numbers only for the variables showing the largest inter-site gaps (e.g., improved flooring, defecation, etc.), while referring to Table 1 for all remaining variables.

- *Thank you. We agree with the reviewer that long lists of variables can be difficult to interpret. We have limited this to factors that vary substantially across sites and are important to STH transmission. We hope this is now more clear.*

- Results p.13, study population. Is it possible to at least use an approximate age for the missing-age residents? Not for the pyramid, but for the age-stratified prevalence estimate in which all adults are in the same bin? Or did you do that? If you did (include them in the adult stratum), please add a sentence about it.

- *The age-weighted prevalence estimate is based only on preschool-age children, school-age children and adults, and any participants with missing ages are excluded. However, given the very small number of missing ages (ranging from 0.0% in India to 0.3% in Malawi) the impact of this is negligible. We have added the following sentence to the Methods:*
  - *"Cluster-specific age-weighted prevalences were calculated based on age distribution in each cluster, excluding infants (who were not eligible to be sampled and therefore had no prevalence data) and those with missing ages."*

- Results, all prevalence estimates. 95% CIs seem somewhat too narrow for the sample size and the additional complexity (stratified sample and clusters). Agresti-Coull intervals are for the plain-vanilla Binomial. Please look into replacing by more realistic intervals that take account of the uncertainty in strata weights, cluster weights, as well as the inter-cluster variability. A bootstrap of some sort might help. Or perhaps some design-effect and delta-method adjustments.

- *We agree with the reviewer that adjustment for design effect is appropriate. The 95% CIs have been replaced with Wilson 95% CIs with a design effect adjustment. However, we do not consider bootstrapping or delta method adjustment appropriate. The baseline prevalence estimates presented are specific to the study area in each site, each of which was fully censused and divided into 40 clusters. All 40 clusters were sampled, so intra-cluster variability does not adversely affect our ability to estimate the prevalence; and as the sample was drawn from the baseline census, the exact strata and cluster weights are known.*
- *We have added a note on the design effect adjustment to the Methods section as follows:*

    "Cluster-specific age-weighted prevalences were calculated based on age distribution in each cluster, and the final prevalence estimates, overall and age-specific, were weighted for cluster size. Precision of site-wide prevalence estimates was adjusted for design effect, and Wilson 95% confidence intervals with design effect adjustment are presented."

- Results, likewise for estimates of k, the CIs seem too narrow in many cases in view of the spread, in particular in Fig. 5B.

- *The 95% confidence intervals reflect the prevalence predicted by the model at a given egg count value. The narrowness of the range is CI ranges in comparison to the data is a reflection of the simple nature of the model being fitted. It excludes other sources of variance, such as uncertainty in the egg count data and variability in the measured egg output for individuals arising from the egg production variability of individual worms and the sensitivity of the Kato Katz diagnostic method. Inclusion of these effects is too complex for the current analysis*

- *The limitations of this simplified model are presented in the supplementary materials as follows:*

> *"The method used here is a simplification and omits two sources of variance which may result in bias. Both arise from the variability in the measured egg count for individuals. These contribute an extra source of uncertainty in both the prevalence and mean egg count in a population. As a result, the method tends to overestimate the aggregation of worms (decrease the value of the aggregation parameter, k). Inclusion of these effects is too complex for the current analysis but has been explored elsewhere (DOI: 10.1186/s13071-019-3686-2)."*

- Figure 5, given the order-of-magnitude difference between India and the others, consider making different y axis scales for India and for the African sites, and making a note of it when referring to the figure in the text. Also, there's a lot of white-space waste (maximum y value too high), particularly in plots A/C/E.

- *We thank the reviewer for the suggestion. We considered this but when viewing these figures side by side with different scale, we were concerned that people may miss the differences and interpret the graphs as more similar than they are. As a result, we would prefer to keep the scale of all y-axes consistent for ease of side-by-side comparison between sites, but are willing to make this change at the Editor's request.*

- **Discussion p.16 line 5. Typo: Fig. 3 is referred to as Fig. 2.**

- *Thank you – the figures were incorrectly labeled in the tables and figures file, with two figures labeled as Figure 3. This has now been corrected.*

The authors report "age-adjusted" prevalence for each site in the abstract and results, but only mention methods for calculating "age-weighted" prevalence. Please ensure consistency in naming convention for prevalence estimates. If estimates were further adjusted by age to compare across sites please specify in the methods. If not please remove "Age-adjusted" terminology as it would indicate that estimates are comparable across sites with respect to similar age-distributions (which it seems is not the case here). Instead, it appears estimates are weighted by age to ensure they reflect the age-distribution within each site.

- *Thank you for alerting us to this error. We've replaced age-adjusted with age-weighted throughout the manuscript.*

Reviewer's Responses to Questions
Review Criteria Required for Acceptance?
As you describe the new analyses required for acceptance, please consider the following:

Methods
-Are the objectives of the study clearly articulated with a clear testable hypothesis stated?
-Is the study design appropriate to address the stated objectives?
-Is the population clearly described and appropriate for the hypothesis being tested?
-Is the sample size sufficient to ensure adequate power to address the hypothesis being tested?
-Were correct statistical analysis used to support conclusions?
-Are there concerns about ethical or regulatory requirements being met?

Reviewer #1: **(No Response)**
Reviewer #2: **This study presents baseline infection data and epidemiological parameters, including host statistics, from a large scale study on three main soil transmitted helminths. The study is informative, in that it covers large areas from three countries, but recapitulates what generally known on the patterns of infection of these three helminths in low/medium income countries.**

This is a very data rich study and it would be useful to look at these data in more detail and address some of the speculations reported in the Discussion. For example, comments are made on a possible effect of gender or sanitation level on the intensity of infection, however, it seems to me that these data are available (table 1) and analyses can be straightforward.

Similarly, it will be useful to examine if there is any spatial pattern in the dynamics of infection and host data, and their relationships, within each country. Are there any high risk areas? And, Are these areas the same for all the three helminths? Given the large variation in some of those trends, I am wondering if this is a consequence of combining the data together.

- *We thank the reviewer for these comments. Owing to differences among the three DeWorm3 study sites and the challenges the reviewer points out with combining the data, separate site-specific publications will explore factors associated with infection at baseline.*

- *Spatial trends are of particular interest to the DeWorm3 team; however, due to the need to maintain blinding during the conduct of the trial, we are not able to publish spatial analyses or maps of prevalence and intensity data until the end of the project. We are happy to provide the blinding guidelines produced in consultation with the trial's Data Safety Monitoring Committee if that would be useful.*

The work needs some attention on formatting, typos, legend description, and particularly, clarify of the sections. In this respect, Methods need to be explained and developed, while it is good to refer to previous studies, a clarity in what is available, from where and when, what is used and how is used in the different analyses will help to move through smoothly. There is some repetition in the text and some sentences seems to

contradict each other. As previous noted, more can be done with the data and Results should be definitely improved. Finally, it will be useful to put this work in a broader context and elaborate more on it in the introduction and discussion.

- *Thank you; we have added explanations on modeling methods (see responses to Reviewer 1 above) and corrected copy editing errors including incorrectly numbered figures.*

Results
-Does the analysis presented match the analysis plan?
-Are the results clearly and completely presented?
-Are the figures (Tables, Images) of sufficient quality for clarity?

Reviewer #1: (No Response)

Reviewer #2: **Please, see above**

Conclusions
-Are the conclusions supported by the data presented?
-Are the limitations of analysis clearly described?
-Do the authors discuss how these data can be helpful to advance our understanding of the topic under study?
-Is public health relevance addressed?

Reviewer #1: (No Response)

Reviewer #2: **Please, see above**

Editorial and Data Presentation Modifications?
Use this section for editorial suggestions as well as relatively minor modifications of existing data that would enhance clarity. If the only modifications needed are minor and/or editorial, you may wish to recommend “Minor Revision” or “Accept”.

Reviewer #1: (No Response)

Reviewer #2: **Please, see above**

Summary and General Comments
Use this section to provide overall comments, discuss strengths/weaknesses of the study, novelty, significance, general execution and scholarship. You may also include additional comments for the author, including concerns about dual publication, research ethics, or publication ethics. If requesting major revision, please articulate the new

experiments that are needed.

Reviewer #1: (No Response)

Reviewer #2: **Please, see above**

PLOS authors have the option to publish the peer review history of their article (what does this mean?). If published, this will include your full peer review and any attached files.

If you choose “no”, your identity will remain anonymous but your review may still be made public.

Do you want your identity to be public for this peer review? For information about this choice, including consent withdrawal, please see our Privacy Policy.

Reviewer #1: Yes: Assaf P. Oron

Reviewer #2: No
